# DDX5 RNA Helicases: Emerging Roles in Viral Infection

**DOI:** 10.3390/ijms19041122

**Published:** 2018-04-09

**Authors:** Wenyu Cheng, Guohua Chen, Huaijie Jia, Xiaobing He, Zhizhong Jing

**Affiliations:** State Key Laboratory of Veterinary Etiological Biology, Key Laboratory of Veterinary Public Health of Agriculture Ministry, Lanzhou Veterinary Research Institute, Chinese Academy of Agricultural Sciences, Lanzhou 730046, Gansu, China; wenyucheng1989@163.com (W.C.); chengguohua78@163.com (G.C.); huaijiejia@163.com (H.J.); hexb835@163.com (X.H.)

**Keywords:** DDX5, RNA helicases, modular domain structure, viral replication, antiviral ability

## Abstract

Asp-Glu-Ala-Asp (DEAD)-box polypeptide 5 (DDX5), also called p68, is a prototypical member of the large ATP-dependent RNA helicases family and is known to participate in all aspects of RNA metabolism ranging from transcription to translation, RNA decay, and miRNA processing. The roles of DDX5 in cell cycle regulation, tumorigenesis, apoptosis, cancer development, adipogenesis, Wnt-β-catenin signaling, and viral infection have been established. Several RNA viruses have been reported to hijack DDX5 to facilitate various steps of their replication cycles. Furthermore, DDX5 can be bounded by the viral proteins of some viruses with unknown functions. Interestingly, an antiviral function of DDX5 has been reported during hepatitis B virus and myxoma virus infection. Thus, the precise roles of this apparently multifaceted protein remain largely obscure. Here, we provide a rapid and critical overview of the structure and functions of DDX5 with a particular emphasis on its role during virus infection.

## 1. Introduction

DEAD-box polypeptide 5 (DDX5), also called p68, is a prototypical member of the large Asp-Glu-Ala-Asp (DEAD)-box ATP-dependent RNA helicases family, which was first identified in 1980 [1]. DDX5 has extensive amino acid sequence homology in various organisms from *Escherichia coli* to humans [2,3,4]. The protein plays multifunctional roles involved in all aspects of RNA metabolism, including translation, splicing, transcription regulation, ribosome biogenesis, mRNA nuclear export, and micro RNA (miRNA) processing [5,6,7,8,9,10,11]. In addition, the functions in DNA modification, cell cycle regulation, tumorigenesis, apoptosis, cancer development, adipogenesis, and nuclear translocation of the Wnt-β-catenin protein through phosphorylation have been established [12,13,14]. In line with the function of the protein in transcription regulation and mRNA splicing, DDX5 has been found to shuttle between the nucleus and the cytoplasm, albeit it predominately localizes in the cell nucleus [15]. Given the crucial roles of DDX5 in RNA biology, several RNA viruses were found to interact with the protein to promote viral replication (Table 1), including severe acute respiratory syndrome (SARS) coronavirus (CoV) [16], human immunodeficiency virus 1 (HIV-1) [17], hepatitis C virus (HCV) [18], Japanese encephalitis virus (JEV) [19], porcine reproductive and respiratory syndrome virus (PRRSV) [20], and influenza virus [21]. Additionally, it has been reported that DDX5 is inhibitory for viral replication for two DNA viruses, hepatitis B virus (HBV) and myxoma virus (MYXV) [22,23]. In view of the importance of DDX5 in regulating virus infection, we here provide a brief and critical overview of the known functions of DDX5, with a particular emphasis on its role during the virus life cycle.

## 2. Modular Domain Structure of DDX5

**Figure 1:**
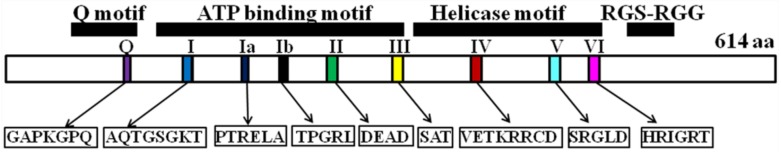
Scheme of DEAD-box polypeptide 5 (DDX5) core architecture and conserved motifs. A schematic representation of DDX5 showing its nine conserved motifs (Q, I, Ia, Ib, II, III, IV, V, and VI) and the corresponding conserved amino acid sequences of motifs are presented. RGS-RGG: Arg-Gly-Ser-Arg-Gly-Gly.

RNA or DNA helicases are categorized based on the substrates they bind, and can be classified into three superfamilies and two families (named SF1 to SF5) based on the occurrences and characteristics of conserved motifs in their primary sequences [26,27]. The DEAD-box helicase family is named for its conserved Asp-Glu-Ala-Asp motif and belongs to the SF2 group [26,27,28], which shares nine conserved motifs shown to be involved in ATPase and helicase activities [29,30,31]. DDX5 is a 68 kD DEAD-box helicase with 614 amino acids in its full length [2]. Consistent with the members of the SF1 and SF2 helicases, DDX5 contains the motifs Q, I, Ia, Ib, II, III, IV, V, and VI, which constitute the core region of its helicase function (Figure 1). The Q motif is named for the conserved glutamine residue that is present in more than 99% of DEAD-box helicase sequences. The Q motif has been found to be necessary for efficient binding of single-stranded RNA (ssRNA) as well as for the conformational changes that are driven by nucleotide binding and ATP hydrolysis [31]. Motif I has been found to be crucial for ATPase and helicase activities through its interaction with motif II and motif III [26,32]. Motifs Ia and Ib participate in RNA-binding and structural rearrangements that occur upon ATP binding and hydrolysis [26,33]. Motifs II and III are necessary for ATPase activity, and they interact with motifs I and VI to collectively form the ATP binding pocket, which leads to the correct positioning of the residues necessary for hydrolysis [26,30]. Motif IV is believed to bind ssRNA and functions as a functional connect ion between motifs IV and V [26]. Motifs V and VI are both important for RNA binding in association with motifs Ia, Ib, and IV [34]. DDX5 also contains a Arg-Gly-Ser-Arg-Gly-Gly (RGS-RGG) motif and an Ile-Gln (IQ) motif, which locate in the C-terminal region. The RGS-RGG motif can function as an RNA-binding site to interact with RNA or DDX3 for modulating biological functions [35].

## 3. DDX5 Is a Host Factor Influencing Viral Infection

Given the ubiquity of RNA helicases in plants and animals and the highly conserved Asp-Glu-x-Asp/His (DExD/H) ATP-binding domain in all helicases, RNA helicases are assumed to play essential roles in a broad array of biological processes, especially RNA metabolism. Growing evidence shows that DDX5 is implicated in the infection of several viruses. By exploiting the driving force of DDX5-mediated ATP hydrolysis or assembling large ribonucleoprotein complexes, RNA viruses can complete their life cycles. However, DDX5 exhibits an antiviral activity during HBV and MYXV infection.

### 3.1. DDX5 Interacts with Severe Acute Respiratory Syndrome Coronavirus Helicase

Severe acute respiratory syndrome coronavirus (SARS-CoV), the causative agent of SARS, was responsible for a large outbreak associated with a 10% fatality rate in 2003 [36,37]. This positive RNA virus encodes a large replicase polyprotein made up of 16 nonstructural proteins (nsp1-16), among which nsp13 is thought to be essential for the virus life cycle [38,39]. As the helicase of SARS-CoV, nsp13 characterizes the enzymatic activities that can unwind both RNA and DNA duplexes [40,41]. Although SARS-CoV carries its own RNA helicases, host RNA helicases may be hijacked by viruses to facilitate the replication of their viral genome; examples include HIV-1 and HCV [42]. Using the SARS-CoV *nsp13* gene as a probe for two-hybrid screening in yeast and mammalian cells, only the *DDX5* gene was identified as interacting with the helicase [16]. Further RNA interference (RNAi) assays confirmed that inhibition of DDX5 results in the suppression of viral replication [16].

Through direct binding to the SARS-CoV helicase, DDX5 may act as a coactivator to enhance viral genome transcription and virus proliferation [16]. Like DDX5, another DEAD-box RNA helicase, DDX1, has been reported to be associated with SARS-CoV and coronavirus infectious bronchitis virus (IBV) nonstructural protein 14 (nsp14) [43]. DDX1 utilizes its C-terminal region, containing motifs V and VI, to interact with nsp14 via the latter’s N-terminal portion. Further manipulation of *DDX1* expression by small interfering RNA and overexpression confirmed that this interaction enhances coronavirus IBV replication [43,44]. Moreover, DDX1 has been identified as a member of the cellular interactomes for the IBV N protein, a nucleocapsid protein encoded by subgenomic mRNAs of coronaviruses [44,45]. Recruitment of DDX1 to the phosphorylated-N-containing complex mediated by N phosphorylation can facilitate template readthrough and longer subgenomic mRNA synthesis [44]. Therefore, the main role of DDX1 and DDX5 when hijacked by coronavirus is to positively modulate viral genome transcription and virus proliferation.

### 3.2. DDX5 Facilitates Human Immunodeficiency Virus 1 mRNA Export

As the causative agent of acquired immune deficiency syndrome (AIDS), human immunodeficiency virus 1 (HIV-1) is a retrovirus of the lentivirus genus with an RNA genome of ~9 kb. Nine polypeptides are encoded by the genome RNA, containing three structural proteins Env (envelope), Gag (group-specific antigen), and Pol (polymerase); four accessory proteins, Nef, Vif, Vpu, and Vpr; and the regulatory proteins, Tat and Rev [46,47]. This “intelligent” pathogen utilizes many host cell factors for its replication. Genome-wide screening technologies have been applied by many groups to clarify host factors that affect HIV-1 replication. More than 300 cellular factors have been identified that may be involved in this process [48,49,50]. Among these factors, several members of the DEAD-box helicase family that act as co-factors to facilitate HIV-1 proliferation, such as RNA helicase A (RHA), DDX1, DDX3, DDX5, DDX10, DDX17, DDX21, DDX28, DHX36, DDX47, DDX52, and DDX56 [17,49,51,52,53,54,55]. Research has revealed that host RNA helicases RHA and DDX3 act as cofactors of Tat and enhance HIV-1 genes expression [56,57,58]. Knockdown of *DDX3* expression or depletion of *DDX3* in cells effectively suppresses the translation of Tat and Rev as well as HIV-1 mRNA transcription [57,58]. DDX3 directly interacts with HIV-1 Tat, which is partially targeted to cytoplasmic stress granules upon DDX3 overexpression or conditions of cell stress, suggesting a potential role of Tat/DDX3 complex in translation. More findings have indicated that DDX3 is recruited to the *trans*-activation responsive (TAR) hairpin by interaction with viral Tat to facilitate HIV-1 mRNA transcription and translation [57,58].

**Figure 2:**
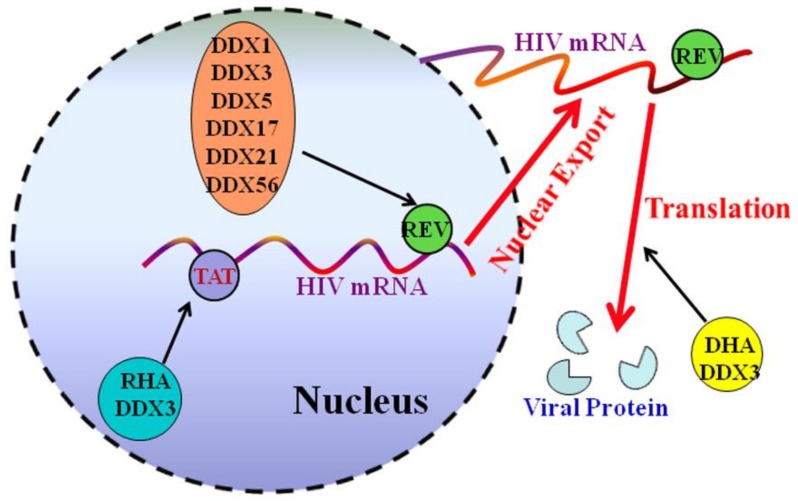
Role of DDX5 and other cellular helicases in the HIV-1 gene expression. DDX5 interacts with HIV Rev and participates in splicing or export of Rev-dependent mRNAs. Other cellular helicases with known functions are involved in HIV mRNA transcription, nuclear export, and translation.

Recently, DDX5 was found to be a new co-factor of HIV-1 that interacted with HIV-1 Rev through the Rev response element (RRE) axis, and thus affects Rev function to enhance HIV-1 replication [17]. Meta-analysis of host cell genes linked to HIV replication showed that DDX5 was involved in Rev-associated complex, suggesting its specific links to HIV-1 replication [50]. DDX5 was confirmed in co-localization with Rev in the nucleus, an interaction that is largely dependent on RNA [17]. Since the main function of Rev is to bind with unspliced and partially spliced HIV-1 transcripts and shuttle them from the nucleus to the cytoplasm [59,60], DDX5 might be a co-factor to bind with RNA and then affect splicing or export of Rev/RRE-dependent mRNAs (Figure 2).

Findings made by Lever’s group showed that DDX5 and DDX17 exist as homo- or heterodimers to be used by HIV but function in different phases of the virus’ lifecycle [55,61]. DDX5 has a phenotype consistent with its involvement in viral transcriptional control, as mentioned above, while it is speculated that DDX17 acts at a later stage, after transcription [61]. Similarly, DDX17 has also been shown to associate with HIV-1 Rev, with helicase knockdown leading to reduced virus release from infected cells [53]. In addition to DDX3, DDX5, and DDX17, other RNA helicases, including RHA, DDX1, DDX21, and DDX56 also have been shown to associate with HIV-1 Rev and to stimulate the Rev function, suggesting distinct DEAD-box RNA helicases could cross-talk to enhance the HIV-1 life cycle at multiple stages through modulating the HIV-1 Rev function (Figure 2).

Compared with other etiological agents, the genome of viruses encodes fewer proteins. Consequently, numerous host factors are harnessed by viruses to complete their life cycles, among which some are pro-viral host factors while others are anti-viral host factors. Viral DNA/RNA replication may be the prime target for the development of efficient and safe novel vaccines or anti-viral therapeutics [62,63,64].

Unlike SARS-CoV, HIV-1 does not encode its own RNA helicase. Taken together, host DDX5 could be a new potential molecular target for the development of anti-viral drugs. There are several studies that focus on specific inhibitors or drugs of the host DEAD-box helicase to inhibit virus replication or treat cancers [65,66,67], but it remains to be determined whether small molecular inhibitors of the interaction between DDX5 and Rev can be found.

### 3.3. DDX5 Is Involved in the Hepatitis C Virus (HCV) Life Cycle

Hepatitis C, a contagious liver disease, is caused by HCV, which belongs to a positive-stranded RNA virus of the family *Flaviviridae*. HCV infects several hundred million people worldwide and results in cirrhosis, steatosis, and hepatocellular carcinoma [68,69]. At least 10 proteins are encoded by the positive single-stranded 9.6 kb RNA genome of HCV, including three structural proteins (the core protein and envelope (E) proteins E1 and E2) and seven nonstructural (NS) proteins (p7, NS2, NS3, NS4A, NS4B, NS5A, and NS5B) [68,69]. As the viral RNA-dependent RNA polymerase (RdRp), NS5B can transcribe the positive- and negative-strand RNA genome of the virus; it has therefore become a common target for antiviral agents [70]. Viral replication requires interaction between RNA, viral, and host proteins [71]. NS5B has been shown to interact with a mass of host proteins through yeast two-hybrid screening, small interfering RNA (siRNA) screening, and transcriptome expression analysis, including T-plastin, eukaryotic initiation factor 4AII, Peptidyl-prolyl isomerase Pin1, and DDX5 [18,72,73,74,75].

Using yeast-two-hybrid screening, DDX5 was identified as an interacting partner of HCV NS5B, one that affects HCV replication [18]. Overexpression of NS5B causes the redistribution of endogenous DDX5 from the nucleus to the cytoplasm; deletion of the C terminal of NS5B abolishes this interaction, while the deletion of the N terminal of NS5B does not influence the distribution of DDX5 [18]. Similarly, another study revealed that DDX5 is redirected from the nucleus to the cytoplasm in Huh7 cells infected with cell culture-produced HCV (HCVcc) [76]. Further experimentation has demonstrated that there are two independent NS5B-binding sites in DDX5: one located at the N-terminus and another at the C-terminus [77]. The first 51 residues of N-terminal fragment are variable, which could fold back to block one of the NS5B binding sites located between 61 and 305 residues in DDX5, suggesting the highly dynamic interaction between DDX5 and NS5B in infected cells [77].

Previous studies have shown that knockdown of DDX5 by RNAi in 293 cells reduced the synthesis of negative-strand HCV RNA, suggesting that DDX5 may be beneficial to the synthesis of the HCV genome (HCV-S1 strain) (Table 2) [18]. Similarly, HCV RNA replication was decreased and the infectivity of HCV (HCV-JFH1 strain) was significantly suppressed in the DDX5 knockdown RSc cells [76]. In line with this observation, the knockdown of *DDX5* in Huh7.5 cells dramatically inhibits HCV replication as determined from the 5′-nontranslated region of the HCV genome [78]. However, contrary results have been shown in Huh7.5 cells by knockdown of the DDX5 through RNAi, in a study that revealed that the level of HCV RNA in the DDX5 siRNA treated cells was higher than in the cells treated with control siRNA, suggesting that DDX5 has the ability to inhibit HCV RNA replication [79]. Further experiments using cell-free infectious virus particles to infect siRNA treated cells showed, curiously, that the increased HCV RNA in the DDX5 siRNA treated cells did not cause increased production of cell-free infectious virus particles (Table 2) [79]. This finding may indicate that DDX5 was involved in a regulatory role at a later stage of the HCV life cycle. DDX5 was also found to be interacted with the 3′-untranslated region (UTR) of HCV, which acts as an enhancer of HCV IRES (internal ribosome entry site) function in hepatic cell lines [80]. The knockdown of *DDX5* results in reduction viral translation efficiency, but has no effects on HCV replicon, suggesting the essential roles of DDX5 in HCV IRES translation [80]. So far, limited focus has been trained on the precise role of DDX5 in HCV replication. Hence, further investigations and studies should aim to elucidate the precise role of DDX5 in HCV replication and determine if DDX5 can interact with other HCV proteins.

### 3.4. DDX5 Acts as a Positive Regulator of Japanese Encephalitis Virus Replication

Japanese encephalitis virus (JEV), a mosquito-borne neurotropic *Flavivirus*, is one of the common causes for epidemic encephalitis in humans and animals. The resulting disease, JE, affects large parts of Asia and the Pacific, where over 3 billion people at risk of exposure to the virus [81,82]. JEV possesses a single-strand positive-sense RNA approximately 11 kb in length, encoding a single polyprotein composed of three structural proteins and seven non-structural (NS) proteins in the order 5′-C-prM-E-NS1-NS2a-NS2b-NS3-NS4a-NS4b-NS5-3′ [83]. Of these, the core (C), premembrane (prM), and envelope (E) proteins are components of extracellular mature virus particles [84]. The seven C-terminal non-structural proteins (NS1–NS5) are involved in multiple steps of viral life cycles such as RNA replication, virus assembly, and innate immunity evasion [83].

Recently, it has been shown that DDX5 can interact with JEV core protein (C), NS3 and NS5, and can host DDX5 act as a positive regulator for JEV replication [19]. Both knockdown of DDX5 and overexpression of DDX5 mutants lacking helicase activity can decrease JEV replication, suggesting that DDX5 and its helicase activity are required for JEV replication [19]. DDX5 binds to the viral 3′-UTR and colocalize with viral RNA, which promotes virus RNA replication but not in viral protein translation. As mentioned above, DDX5 has an interaction relationship with 3′-UTR of HCV and plays a role in HCV IRES translation. Similarly, DDX5 might participate in the translation of JEV IRES. The interaction between DDX5 and the three viral proteins (C, NS3, and NS5) co-localized with viral RNA in the cytoplasm during infection might facilitate the unwinding the intermediate RNA duplexes [19]. The C protein of JEV was found to localize in both the cytoplasm and the nucleus; the mature C protein, released from the endoplasmic reticulum (ER) membrane, is believed to bind to the genomic RNA [85]. Kinetic study of viral RNA and protein syntheses have shown that, following translation at the early step of infection, it was translocated into the nucleus, and then facilitated RNA replication at the late phase of infection. NS3 functions as RNA helicase, RNA triphosphatase, and RNA-stimulated nucleoside triphosphatase. NS5 works as RNA-dependent RNA polymerase and methyltransferase [86]. These two viral proteins (NS3 and NS5), together with NS2a, specifically bind the 3′-untranslated region and lead to the formation of replication complex (RC) [87]. Host DDX5 is recruited by the viral proteins and binds to this RC, and then utilizes its helicase activity to regulate JEV replication [19]. Since viral RNA replication is a quite dynamic process, DDX5 shuttles between nucleus and cytoplasm to increase the efficiency of RNA separation [19].

In addition to DDX5, helicase DDX3 was also found to be involved in JEV replication [88]. Viral NS3 and NS5 may interact with DDX3, which binds to JEV 5′- and 3′-untranslated regions to positively regulate viral RNA translation. When using chemical compounds (Cmp6 and Cmp8) to inhibit the helicase activity of DDX3 in HIV and JEV infected cells, viral RNA replication was remarkably inhibited [65,88]. However, no drugs or molecule inhibitors targeting DDX5 are available. Therefore, DDX5 could be a novel target for the development of anti-viral drugs.

Besides HCV and JEV, other members of the *Flaviviridae* family were also found to hijack DDX5 during virus infection. The genus *Pestivirus*, a group of small positive-stranded RNA viruses, belongs to the family *Flavivirus*, which cause economically important diseases of farm animals and includes bovine viral diarrhea virus (BVDV), classical swine fever virus (CSFV), and border disease virus (BDV) [89]. The enveloped pestiviruses contain single-stranded RNA genomes of approximately 12.3–12.5 kb that consists of a single open reading frame, encoding only 12 proteins. These are co- and post-translationally processed from a single RNA into four structural and eight nonstructural proteins in the order NH2-N^pro^-C-E^rns^-E1-E2-p7-NS2-NS3-NS4A-NS5B-COOH [90,91]. N^pro^, named for the viral N-terminal protease, is a 168-amino-acid autoprotease, with cysteine protease activity in a Glu22-His49-Cys69 triad that acts to cleave itself from the nascent polypeptide [92]. Early work using mass spectrometry showed that N^pro^ binds to DDX5, and also to DDX3X and DHX9 [25]. The distribution of N^pro^ in ribosomal and ribonucleoprotein particles, as well as its interactions with several RNA helicases, suggest that N^pro^ is involved with virus RNA translation; DDX5 possibly contributes to this process. Nevertheless, the function of DDX5 in modulation of viral replication remains to be determined.

### 3.5. DDX5 Positively Regulates the Replication of Porcine Reproductive and Respiratory Syndrome Virus

Porcine reproductive and respiratory syndrome virus (PRRSV), the causative agent of one of the most economically important global swine diseases, is classified in the genus *Arterivirus* of the family *Arteriviridae* [93]. PRRSV contains a single-strand, positive-sense RNA of approximately 15 kb in length, composed of at least 10 open reading frames (ORFs), and a poly-A tail at the 3′-terminus [94]. ORF1a and ORF1b encode the replication-related polymerase proteins and are processed into at least 13 nonstructural proteins (nsps) by self-cleavage [95].

Among the nsps encoded by the ORF1b region of PRRSV, nsp9 is considered to be involved in viral replication and genomic transcription [96]. This protein possesses a RNA-dependent RNA polymerase (RdRp) domain in its C-terminal portion that contributes to its polymerase activity and the virulence of PRRSV [97]. Using the yeast two-hybrid method, the host DDX5 was found to interact with the nsp9 of PRRSV [20]. Overexpression of DDX5 in MARC-145 cells showed an enhancement effect on the replication of PRRSV; meanwhile, silencing of DDX5 expression in MARC-145 cells might significantly inhibit the replication of virus particles, suggesting that DDX5 might function as a cellular cofactor that positively regulates PRRSV replication [20].

Confocal immunofluorescence assay has revealed that DDX5 co-localizes with nsp9 in the cytoplasm with a perinuclear pattern in HEK293 cells, MARC-145 cells, and the PAM cell line, suggesting that endogenous DDX5 might diffuse from nucleus to cytoplasm and help to unwind the viral RNA through its helicase activity. This hypothesis was confirmed by co-immunoprecipitation (Co-IP) assay, which showed that the DEAD and helicase domains (aa1-436) of DDX5 bind with the C-terminal portion of nsp9 [20]. Interestingly, PRRSV carries its own helicase, nsp10, with helicase activity; the recruitment of host DDX5 by nsp9 may function as a cofactor for RNA unwinding, export, and/or translation of the PRRSV during viral genomic replication and transcription [20,96].

### 3.6. DDX5 Enhances the Polymerase Activity of Influenza Virus

Influenza A virus is a major public health problem, causing seasonal epidemics in humans and occasionally lethal global pandemics, as happened in 1918–1919 (H1N1), 1957 (H2N2), 1968 (H3N2), and 2009 (swine-origin H1N1) [98]. Currently, the H5N1 subtype circulating in wild and domestic birds is capable of crossing the species barrier into humans and constitutes huge threats to both animals and public health [99]. As many as 500 million people a year are infected with influenza A virus worldwide, with more than 500,000 deaths [100]. Characterized by its high potential for mutations and adaptations, influenza virus presents a significant challenge to disease control and the pharmaceutical industry [101].

It has been demonstrated that the trimeric viral polymerase (a complex consisting of the PB1, PB2, and PA subunits) and nucleoprotein (NP) contribute to the pathogenicity of H5N1 viruses in humans and other species of mammals [102]. Functional genomics and proteomics approaches have identified a number of human proteins that associate with viral polymerases, including DDX5 and DDX17 [21]. The required role of DDX5 and DDX17 in H5N1 virus replication has led to a significant reduction in the virus titer after knockdown of these proteins [21]. It has been observed that DDX5 colocalizes with viral NPs in the nucleus during viral RNA replication and transcription. Likewise, DDX17 has been found to colocalize with H5N1 NP. However, the predominant distribution of DDX17 in uninfected cells is in the nucleus, where DDX17 exhibits as punctated [21], whereas, in H5N1 infected cells, DDX17 was shown to be relocated in the cytoplasm, a change that exhibits its essential roles in efficient H5N1 mRNA and viral RNA (vRNA) synthesis in both human and avian cells. Given the multiple functions of DDX5 and DDX17 in ribosomal pre-rRNA (ribosomal RNA) processing and rRNA nuclear export [103], we speculate that influenza viruses have evolved to hijack host DDX5 and/or DDX17 in order to facilitate their own proteins’ nuclear export.

### 3.7. DDX5 Interacts with EBNA2 of Epstein-Barr Virus

Epstein-Barr virus (EBV), a type of oncogenic human herpesvirus, is ubiquitous, causing over 90% infection in the exposed human population [104]. Infections cause no symptoms in young children but result in the development of infectious mononucleosis at adolescence or later [105]. As with other herpesvirus, EBV possesses a 170 kb double-stranded DNA genome encoding different sets of viral proteins and microRNAs during lytic and latent infection. Epstein-Barr virus nuclear antigen 2 (EBNA2) is a latency type III expressed protein that interacts with various types of host cell proteins to regulate cellular and viral gene transcription [106]. Recent work using immunoprecipitation identified DDX5 as an interacting partner of EBNA2 [24]. As both EBNA2 and DDX5 contain RG-repeat elements and mono-methylated arginine (MMA) residues [107], it has been proposed that DDX5 probably serves as methylation substrate to facilitate gene expression activation by EBNA2. However, the relevance of EBNA2-DDX5 interaction and functions during virus infection still remains elusive, and more studies are needed.

### 3.8. DDX5 Restricts Myxoma Virus Replication and HBV Transcription

Being a member of the *Leporipoxvirus* genus, Myxoma virus exhibits a specific rabbit-restricted host tropism but reveals a much broader cellular host range in cultured cells [108]. MYXV has been shown to selectively infect a variety of human cancer cell lines derived from a diverse group of tissues, leading to study of MYXV as a potential oncolytic virotherapeutic for various classes of human cancer [109]. Recently, a siRNA library screen targeting the 58 human DEAD-box RNA helicases has shown that DDX5 knockdown enhances MYXV replication. The knockdown of DDX5 in multiple human cell lines increased MYXV replication and enhanced foci size and virus spread, suggesting its potential roles in antiviral responses or regulation of innate immune responses [22]. In addition, DDX5 has shown increased expression in human monocyte-derived dendritic cells infected with attenuated strains of vaccinia virus (VACV) [110]. However, the exact function of this molecule in VACV infection was not established in that study. Unlike MYXV, bunyavirus replication is unaffected by DDX5 silencing, so that the antiviral function of DDX5 remains undefined, as DDX5 depletion upregulated DDX17 expression, restricting bunyavirus infection in an interferon-independent manner [111].

Additionally, DDX5 has a role in transcription of HBV minichromosome by a mechanism not yet determined [23]. HBV is also the causative agent of chronic hepatitis, which progresses to liver cirrhosis and hepatocellular carcinoma (HCC) worldwide [112,113]. Although HBV is a DNA virus, the replication of its DNA genome depends on reverse transcription [112]. After entry, the HBV DNA is transported into the nucleus and is converted into covalently closed circular (ccc) DNA [114], which can assume a minichromosome-like structure and acts as the template for transcription of four viral RNAs [114]. Recently, it was shown that DDX5, by regulating PRC2 (Polycomb repressive complex 2) stability and function, represses HBV minichromosome and the expression of specific host genes involved in HBV-mediated hepatocarcinogenesis [23]. DDX5 interacts with and stabilizes suppressor of zeste 12 homolog (SUZ12), which is an essential subunit of PRC2 [23,115]. As an expression of viral protein HBx in HBV-infected cells, DDX5 was downregulated and leads to the degradation of SUZ12, which results in the transcription viral cccDNA-encoded genes [23]. Moreover, DDX5 expression was found to be reduced in a group of HBV-associated HCCs and this can be an important molecule in pathogenesis of poor prognosis HBV-mediated liver cancer [23].

In recent years, as the emerging roles of some RNA helicases in host innate response have been established, studies have shown that viral nucleic acids are sensed by DDX1, DHX9, DDX21, DHX33, DHX36, DDX41, retinoic acid-inducible gene 1 (RIG-I/DDX58), and melanoma differentiation- associated gene 5 (MDA-5), leading to type I interferon production and an antiviral response [116,117,118,119,120,121]. These helicases (RIG-I and MDA-5) are characterized by caspase activation and recruitment domains (CARD) at the N-terminal region, which interact with other CARD-containing proteins to transduce the signaling cascade [122]. Alternatively, the functions in nucleic acids recognition of helicases—such as DDX1, DHX9, DDX21, DHX33, DHX36 and DDX41—which are lacking in CARD, have all been established in certain human plasmacytoid dendritic cells [116,117,118,119,120]. DDX5 lacks the CARD signaling domains, and no literature indicates the function of DDX5 in regulating the production of type I interferon. The issue of whether DDX5 contributes to antiviral responses or regulation of innate immune responses remains uncertain, and more studies are needed.

## 4. Conclusions

As summarized in this review, the multifaceted protein DDX5 has been shown to be involved in RNA metabolism and viral infection, especially for RNA viruses. It is intriguing that DDX5 is the target of manipulation by different viruses. Each RNA virus seems to hijack host DDX5 in order to facilitate its own replication. With conserved ATP binding motif and helicase motif, DDX5 utilizes the free energy to change of binding and hydrolyzing a nucleotide triphosphate to dissociate duplexes or displace bound proteins, which, on one hand, DDX5 may translocate along with viral DNA/RNA; on the other hand, DDX5 may unwind double-stranded regions to promote the expression of viral proteins [26]. However, DDX5 exhibits a role in antiviral responses during HBV and MYXV infection. This might indicate that a requirement of DDX5 for viral replication is a more universal feature of RNA viruses. The opposite roles of DDX5 between DNA and RNA infection likely reflect the different modes of the biosynthesis of RNA and DNA viruses. As mentioned, the using of DDX5 for viral replication could be a therapeutic target for development of antiviral drugs.

Even though emerging studies on DDX5 have supplemented our knowledge about the multiple functions of DDX5 in recent years, the overall picture still remains fragmentary. Research is still needed to resolve seemingly contradictory data on the role(s) of DDX5 in response to different virus. As conserved motifs in the primary sequence, DExD/H helicases are highly conserved from viruses and bacteria to humans, and these proteins have overlapping functions. In addition, these proteins generally act as components of large multi-protein complexes, with each other or other factors, to regulate transcription. Therefore, studies should also attempt to establish whether the effects of DDX5 on viral replication after knockdown are unique or whether other helicases supplement abolished function via small interfering RNA.

## Figures and Tables

**Table 1:** Overview of the role of DEAD box polypeptide 5 (DDX5) in virus infection.

Virus	Viral Binding Protein	Effect of DDX5 on Viral Replication	Ref.
SARS-CoV	nsp13	Enhances virus proliferation	[16]
IBV-CoV	nsp13	Enhances virus proliferation	[16]
HIV-1	Rev	Enhances the Rev-dependent RNA export	[17]
HCV	NS5B	Required for transcription of viral RNA	[18]
JEV	C, NS3 and NS5	Required for viral RNA replication	[19]
PRRSV	nsp9	Required for viral replication	[20]
Influenza virus	NP	Required for viral replication	[21]
EBV	EBNA2	Unknown	[24]
*Pestivirus*	N^pro^	Unknown	[25]
Myxoma virus	Unknown	Inhibits viral replication	[22]
HBV	Unknown	Inhibits viral replication	[23]

SARS-CoV: severe acute respiratory syndrome coronavirus; IBV-CoV: infectious bronchitis virus coronavirus; HIV-1: human immunodeficiency virus 1; HBV: hepatitis B virus; HCV: hepatitis C virus; JEV: japanese encephalitis virus; PRRSV: porcine reproductive and respiratory syndrome virus; EBV: Epstein-Barr virus; nsp: nonstructural protein; NP: nucleoprotein; EBNA2: Epstein–Barr virus nuclear antigen 2; N^pro^: N-terminal protease.

**Table 2:** The effects on HCV RNA and particles after the knockdown of DDX5 in different cell lines.

Cell Lines	HCV Strains	Effect on HCV RNA	Effect on HCV Particles	Ref.
293	HCV-S1	Reduced	Unknown	[18]
Huh7.5	J6/JFH-1	Increased	Promotes a late step in viral replication	[81]
Huh-7-NS3-3′	Unknown	Reduced	Unknown	[80]
RSc cells	HCV-JFH1	Reduced	Suppressed	[78]

## References

[1] Lane D.P., Hoeffler W.K. (1980). SV40 large T shares an antigenic determinant with a cellular protein of molecular weight 68,000. Nature.

[2] Hirling H., Scheffner M., Restle T., Stahl H. (1989). RNA helicase activity associated with the human p68 protein. Nature.

[3] Iggo R., Picksley S., Southgate J., McPheat J., Lane D.P. (1990). Identification of a putative RNA helicase in *E. coli*. Nucleic Acids Res..

[4] Iggo R.D., Jamieson D.J., MacNeill S.A., Southgate J., McPheat J., Lane D.P. (1991). 68 RNA helicase: Identification of a nucleolar form and cloning of related genes containing a conserved intron in yeasts. Mol. Cell Biol..

[5] Watanabe M., Yanagisawa J., Kitagawa H., Takeyama K., Ogawa S., Arao Y., Suzawa M., Kobayashi Y., Yano T., Yoshikawa H. (2001). A subfamily of RNA-binding DEAD-box proteins acts as an estrogen receptor α coactivator through the N-terminal activation domain (AF-1) with an RNA coactivator, SRA. EMBO J..

[6] Liu Z.R. (2002). p68 RNA helicase is an essential human splicing factor that acts at the U1 snRNA-5′ splice site duplex. Mol. Cell Biol..

[7] Wilson B.J., Bates G.J., Nicol S.M., Gregory D.J., Perkins N.D., Fuller-Pace F.V. (2004). The p68 and p72 DEAD-box RNA helicases interact with HDAC1 and repress transcription in a promoter-specific manner. BMC Mol. Biol..

[8] Lin C., Yang L., Yang J.J., Huang Y., Liu Z.R. (2005). ATPase/helicase activities of p68 RNA helicase are required for pre-mRNA splicing but not for assembly of the spliceosome. Mol. Cell Biol..

[9] Jalal C., Uhlmann-Schiffler H., Stahl H. (2007). Redundant role of DEAD-box proteins p68 (DDX5) and p72/p82 (Ddx17) in ribosome biogenesis and cell proliferation. Nucleic Acids Res..

[10] Zonta E., Bittencourt D., Samaan S., Germann S., Dutertre M., Auboeuf D. (2013). The RNA helicase DDX5/p68 is a key factor promoting c-fos expression at different levels from transcription to mRNA export. Nucleic Acids Res..

[11] Dardenne E., Espinoza M.P., Fattet L., Germann S., Lambert M.P., Neil H., Zonta E., Mortada H., Gratadou L., Deygas M. (2014). RNA helicases DDX5 and DDX17 dynamically orchestrate transcription, miRNA, and splicing programs in cell differentiation. Cell Rep..

[12] Saporita A.J., Chang H.C., Winkeler C.L., Apicelli A.J., Kladney R.D., Wang J., Townsend R.R., Michel L.S., Weber J.D. (2011). RNA helicase DDX5 is a p53-independent target of ARF that participates in ribosome biogenesis. Cancer Res..

[13] Jost J.P., Schwarz S., Hess D., Angliker H., Fuller-Pace F.V., Stahl H., Thiry S., Siegmann M. (1999). A chicken embryo protein related to the mammalian DEAD-box protein p68 is tightly associated with the highly purified protein-RNA complex of 5-MeC-DNA glycosylase. Nucleic Acids Res..

[14] Yang L., Lin C., Liu Z.R. (2005). Phosphorylations of DEAD-box p68 RNA helicase are associated with cancer development and cell proliferation. Mol. Cancer Res..

[15] Wang H., Gao X., Huang Y., Yang J., Liu Z.R. (2009). P68 RNA helicase is a nucleocytoplasmic shuttling protein. Cell Res..

[16] Chen J.Y., Chen W.N., Poon K.M., Zheng B.J., Lin X., Wang Y.X., Wen Y.M. (2009). Interaction between SARS-CoV helicase and a multifunctional cellular protein (DDX5) revealed by yeast and mammalian cell two-hybrid systems. Arch. Virol..

[17] Zhou X., Luo J., Mills L., Wu S., Pan T., Geng G., Zhang J., Luo H., Liu C., Zhang H. (2013). DDX5 facilitates HIV-1 replication as a cellular co-factor of Rev. PLoS ONE.

[18] Goh P.Y., Tan Y.J., Lim S.P., Tan Y.H., Lim S.G., Fuller-Pace F., Hong W. (2004). Cellular RNA helicase p68 relocalization and interaction with the hepatitis C virus (HCV) NS5B protein and the potential role of p68 in HCV RNA replication. J. Virol..

[19] Li C., Ge L.L., Li P.P., Wang Y., Sun M.X., Huang L., Ishag H., Di D.D., Shen Z.Q., Fan W.X. (2013). The DEAD-box RNA helicase DDX5 acts as a positive regulator of Japanese encephalitis virus replication by binding to viral 3′ UTR. Antivir. Res..

[20] Zhao S., Ge X., Wang X., Liu A., Guo X., Zhou L., Yu K., Yang H. (2015). The DEAD-box RNA helicase 5 positively regulates the replication of porcine reproductive and respiratory syndrome virus by interacting with viral Nsp9 in vitro. Virus Res..

[21] Bortz E., Westera L., Maamary J., Steel J., Albrecht R.A., Manicassamy B., Chase G., Martínez-Sobrido L., Schwemmle M., García-Sastre A. (2011). Host- and strain-specific regulation of influenza virus polymerase activity by interacting cellular proteins. MBio.

[22] Rahman M.M., Bagdassarian E., Ali M.A.M., McFadden G. (2017). Identification of host DEAD-box RNA helicases that regulate cellular tropism of oncolytic Myxoma virus in human cancer cells. Sci. Rep..

[23] Zhang H., Xing Z., Mani S.K., Bancel B., Durantel D., Zoulim F., Tran E.J., Merle P., Andrisani O. (2016). RNA helicase DEAD-box protein 5 regulates Polycomb repressive complex 2/Hox transcript antisense intergenic RNA function in hepatitis B virus infection and hepatocarcinogenesis. Hepatology.

[24] Ayoubian H., Fröhlich T., Pogodski D., Flatley A., Kremmer E., Schepers A., Feederle R., Arnold G.J., Grässer F.A. (2017). Antibodies against the mono-methylated arginine-glycine repeat (MMA-RG) of the Epstein-Barr virus nuclear antigen 2 (EBNA2) identify potential cellular proteins targeted in viral transformation. J. Gen. Virol..

[25] Jefferson M., Donaszi-Ivanov A., Pollen S., Dalmay T., Saalbach G., Powell P.P. (2014). Host factors that interact with the *Pestivirus* N-terminal protease, N^pro^, are components of the ribonucleoprotein complex. J. Virol..

[26] Cordin O., Banroques J., Tanner N.K., Linder P. (2006). The DEAD-box protein family of RNA helicases. Gene.

[27] Linder P., Fuller-Pace F. (2015). Happy birthday: 25 years of DEAD-box proteins. Methods Mol. Biol..

[28] Caruthers J.M., McKay D.B. (2002). Helicase structure and mechanism. Curr. Opin. Struct. Biol..

[29] Tanner N.K. (2003). The newly identified Q motif of DEAD-box helicases is involved in adenine recognition. Cell Cycle.

[30] Rocak S., Linder P. (2004). DEAD-box proteins: The driving forces behind RNA metabolism. Nat. Rev. Mol. Cell Biol..

[31] Cordin O., Tanner N.K., Doère M., Linder P., Banroques J. (2004). The newly discovered Q motif of DEAD-box RNA helicases regulates RNA-binding and helicase activity. EMBO J..

[32] Tanner N.K., Linder P. (2001). DExD/H box RNA helicases: From generic motors to specific dissociation functions. Mol. Cell.

[33] Schwer B., Meszaros T. (2000). RNA helicase dynamics in pre-mRNA splicing. EMBO J..

[34] Schütz P., Karlberg T., van den Berg S., Collins R., Lehtiö L., Högbom M., Holmberg-Schiavone L., Tempel W., Park H.W., Hammarström M. (2010). Comparative structural analysis of human DEAD-box RNA helicases. PLoS ONE.

[35] Choi Y.J., Lee S.G. (2012). The DEAD-box RNA helicase DDX3 interacts with DDX5, co-localizes with it in the cytoplasm during the G2/M phase of the cycle, and affects its shuttling during mRNP export. J. Cell Biochem..

[36] Decroly E., Debarnot C., Ferron F., Bouvet M., Coutard B., Imbert I., Gluais L., Papageorgiou N., Sharff A., Bricogne G. (2011). Crystal structure and functional analysis of the SARS-coronavirus RNA cap 2′-*O*-methyltransferase nsp10/nsp16 complex. PLoS Pathog..

[37] Bouvet M., Imbert I., Subissi L., Gluais L., Canard B., Decroly E. (2012). RNA 3′-end mismatch excision by the severe acute respiratory syndrome coronavirus nonstructural protein nsp10/nsp14 exoribonuclease complex. Proc. Natl. Acad. Sci. USA.

[38] Thiel V., Ivanov K.A., Putics A., Hertzig T., Schelle B., Bayer S., Weissbrich B., Snijder E.J., Rabenau H., Doerr H.W. (2003). Mechanisms and enzymes involved in SARS coronavirus genome expression. J. Gen. Virol..

[39] Adedeji A.O., Marchand B., Te Velthuis A.J., Snijder E.J., Weiss S., Eoff R.L., Singh K., Sarafianos S.G. (2012). Mechanism of nucleic acid unwinding by SARS-CoV helicase. PLoS ONE.

[40] Lee N.R., Kwon H.M., Park K., Oh S., Jeong Y.J., Kim D.E. (2010). Cooperative translocation enhances the unwinding of duplex DNA by SARS coronavirus helicase nsp13. Nucleic Acids Res..

[41] Subissi L., Imbert I., Ferron F., Collet A., Coutard B., Decroly E., Canard B. (2014). SARS-CoV ORF1b-encoded nonstructural proteins 12–16: Replicative enzymes as antiviral targets. Antivir. Res..

[42] Kwong A.D., Rao B.G., Jeang K.T. (2005). Viral and cellular RNA helicases as antiviral targets. Nat. Rev. Drug Discov..

[43] Xu L., Khadijah S., Fang S., Wang L., Tay F.P., Liu D.X. (2010). The cellular RNA helicase DDX1 interacts with coronavirus nonstructural protein 14 and enhances viral replication. J. Virol..

[44] Wu C.H., Chen P.J., Yeh S.H. (2014). Nucleocapsid phosphorylation and RNA helicase DDX1 recruitment enables coronavirus transition from discontinuous to continuous transcription. Cell Host Microbe.

[45] Emmott E., Munday D., Bickerton E., Britton P., Rodgers M.A., Whitehouse A., Zhou E.M., Hiscox J.A. (2013). The cellular interactome of the coronavirus infectious bronchitis virus nucleocapsid protein and functional implications for virus biology. J. Virol..

[46] Carter C.A., Ehrlich L.S. (2008). Cell biology of HIV-1 infection of macrophages. Annu. Rev. Microbiol..

[47] Faust T.B., Binning J.M., Gross J.D., Frankel A.D. (2017). Making sense of multifunctional proteins: Human immunodeficiency virus type 1 accessory and regulatory proteins and connections to transcription. Annu. Rev. Virol..

[48] Fellay J., Shianna K.V., Ge D., Colombo S., Ledergerber B., Weale M., Zhang K., Gumbs C., Castagna A., Cossarizza A. (2007). A whole-genome association study of major determinants for host control of HIV-1. Science.

[49] Brass A.L., Dykxhoorn D.M., Benita Y., Yan N., Engelman A., Xavier R.J., Lieberman J., Elledge S.J. (2008). Identification of host proteins required for HIV infection through a functional genomic screen. Science.

[50] Bushman F.D., Malani N., Fernandes J., D’Orso I., Cagney G., Diamond T.L., Zhou H., Hazuda D.J., Espeseth A.S., König R. (2009). Host cell factors in HIV replication: Meta-analysis of genome-wide studies. PLoS Pathog..

[51] Fang J., Kubota S., Yang B., Zhou N., Zhang H., Godbout R., Pomerantz R.J. (2004). A DEAD-box protein facilitates HIV-1 replication as a cellular co-factor of Rev. Virology.

[52] Jeang K.T., Yedavalli V. (2006). Role of RNA helicases in HIV-1 replication. Nucleic Acids Res..

[53] Yasuda-Inoue M., Kuroki M., Ariumi Y. (2013). Distinct DDX DEAD-box RNA helicases cooperate to modulate the HIV-1 Rev function. Biochem. Biophys. Res. Commun..

[54] Ariumi Y. (2014). Multiple functions of DDX3 RNA helicase in gene regulation, tumorigenesis, and viral infection. Front. Genet..

[55] Williams C.A., Abbink T.E., Jeang K.T., Lever A.M. (2015). Identification of RNA helicases in human immunodeficiency virus 1 (HIV-1) replication—A targeted small interfering RNA library screen using pseudotyped and WT HIV-1. J. Gen. Virol..

[56] Fujii R., Okamoto M., Aratani S., Oishi T., Ohshima T., Taira K., Baba M., Fukamizu A., Nakajima T. (2001). A role of RNA helicase A in cis-acting transactivation response element-mediated transcriptional regulation of human immunodeficiency virus type 1. J. Biol. Chem..

[57] Lai M.C., Wang S.W., Cheng L., Tarn W.Y., Tsai S.J., Sun H.S. (2013). Human DDX3 interacts with the HIV-1 Tat protein to facilitate viral mRNA translation. PLoS ONE.

[58] Yasuda-Inoue M., Kuroki M., Ariumi Y. (2013). DDX3 RNA helicase is required for HIV-1 Tat function. Biochem. Biophys. Res. Commun..

[59] Rausch J.W., Grice S.F. (2015). HIV rev assembly on the rev response element (RRE): A structural perspective. Viruses.

[60] Sherpa C., Rausch J.W., Le Grice S.F., Hammarskjold M.L., Rekosh D. (2015). The HIV-1 Rev response element (RRE) adopts alternative conformations that promote different rates of virus replication. Nucleic Acids Res..

[61] Sithole N., Williams C., Vaughan A., Lever A. (2015). The roles of DEAD-box helicases in the life cycle of HIV-1. Lancet.

[62] Moss B. (2013). Poxvirus DNA replication. Cold Spring Harb. Perspect. Biol..

[63] Gehring A., Bertoletti A., Tavis J.E. (2014). Host factor-targeted hepatitis B virus therapies. Intervirology.

[64] König R., Stertz S. (2015). Recent strategies and progress in identifying host factors involved in virus replication. Curr. Opin. Microbiol..

[65] Radi M., Falchi F., Garbelli A., Samuele A., Bernardo V., Paolucci S., Baldanti F., Schenone S., Manetti F., Maga G. (2012). Discovery of the first small molecule inhibitor of human DDX3 specifically designed to target the RNA binding site: Towards the next generation HIV-1 inhibitors. Bioorg. Med. Chem. Lett..

[66] Samal S.K., Routray S., Veeramachaneni G.K., Dash R., Botlagunta M. (2015). Ketorolac salt is a newly discovered DDX3 inhibitor to treat oral cancer. Sci. Rep..

[67] Bol G.M., Vesuna F., Xie M., Zeng J., Aziz K., Gandhi N., Levine A., Irving A., Korz D., Tantravedi S. (2015). Targeting DDX3 with a small molecule inhibitor for lung cancer therapy. EMBO Mol. Med..

[68] McGivern D.R., Lemon S.M. (2011). Virus-specific mechanisms of carcinogenesis in hepatitis C virus associated liver cancer. Oncogene.

[69] Kao C.C., Yi G., Huang H.C. (2016). The core of hepatitis C virus pathogenesis. Curr. Opin. Virol..

[70] Xie Y., Ogah C.A., Jiang X., Li J., Shen J. (2016). Nucleoside inhibitors of Hepatitis C virus NS5B polymerase: A systematic review. Curr. Drug Targets.

[71] Rosenberg S. (2001). Recent advances in the molecular biology of hepatitis C virus. J. Mol. Biol..

[72] Gao L., Tu H., Shi S.T., Lee K.J., Asanaka M., Hwang S.B., Lai M.M. (2003). Interaction with a ubiquitin-like protein enhances the ubiquitination and degradation of hepatitis C virus RNA-dependent RNA polymerase. J. Virol..

[73] Lim Y.S., Tran H.T., Park S.J., Yim S.A., Hwang S.B. (2011). Peptidyl-prolyl isomerase Pin1 is a cellular factor required for hepatitis C virus propagation. J. Virol..

[74] Pham L.V., Ngo H.T., Lim Y.S., Hwang S.B. (2012). Hepatitis C virus non-structural 5B protein interacts with cyclin A2 and regulates viral propagation. J. Hepatol..

[75] Yoo Y.H., Yun J., Yoon C.N., Lee J.S. (2015). Chemical proteomic identification of T-plastin as a novel host cell response factor in HCV infection. Sci. Rep..

[76] Kuroki M., Ariumi Y., Hijikata M., Ikeda M., Dansako H., Wakita T., Shimotohno K., Kato N. (2013). PML tumor suppressor protein is required for HCV production. Biochem. Biophys. Res. Commun..

[77] Dutta S., Gupta G., Choi Y.W., Kotaka M., Fielding B.C., Song J., Tan Y.J. (2012). The variable N-terminal region of DDX5 contains structural elements and auto-inhibits its interaction with NS5B of hepatitis C virus. Biochem. J..

[78] Harris D., Zhang Z., Chaubey B., Pandey V.N. (2006). Identification of cellular factors associated with the 3′-nontranslated region of the hepatitis C virus genome. Mol. Cell Proteom..

[79] Upadya M.H., Aweya J.J., Tan Y.J. (2014). Understanding the interaction of hepatitis C virus with host DEAD-box RNA helicases. World J. Gastroenterol..

[80] Ríos-Marco P., Romero-López C., Berzal-Herranz A. (2016). The cis-acting replication element of the Hepatitis C virus genome recruits host factors that influence viral replication and translation. Sci. Rep..

[81] Yun S.I., Lee Y.M. (2014). Japanese encephalitis: The virus and vaccines. Hum. Vaccines Immunother..

[82] Heffelfinger J.D., Li X., Batmunkh N., Grabovac V., Diorditsa S., Liyanage J.B., Pattamadilok S., Bahl S., Vannice K.S., Hyde T.B. (2017). Japanese encephalitis surveillance and immunization-Asia and Western Pacific regions, 2016. MMWR Morb. Mortal. Wkly. Rep..

[83] Selisko B., Wang C., Harris E., Canard B. (2014). Regulation of *Flavivirus* RNA synthesis and replication. Curr. Opin. Virol..

[84] Kofler R.M., Heinz F.X., Mandl C.W. (2002). Capsid protein C of tick-borne encephalitis virus tolerates large internal deletions and is a favorable target for attenuation of virulence. J. Virol..

[85] Katoh H., Okamoto T., Fukuhara T., Kambara H., Morita E., Mori Y., Kamitani W., Matsuura Y. (2013). Japanese encephalitis virus core protein inhibits stress granule formation through an interaction with Caprin-1 and facilitates viral propagation. J. Virol..

[86] Egloff M.P., Benarroch D., Selisko B., Romette J.L., Canard B. (2002). An RNA cap (nucleoside-2′-*O*-)-methyltransferase in the *Flavivirus* RNA polymerase NS5: Crystal structure and functional characterization. EMBO J..

[87] Uchil P.D., Satchidanandam V. (2003). Architecture of the flaviviral replication complex. Protease, nuclease, and detergents reveal encasement within double-layered membrane compartments. J. Biol. Chem..

[88] Li C., Ge L.L., Li P.P., Wang Y., Dai J.J., Sun M.X., Huang L., Shen Z.Q., Hu X.C., Ishag H. (2014). Cellular DDX3 regulates Japanese encephalitis virus replication by interacting with viral un-translated regions. Virology.

[89] Peterhans E., Schweizer M. (2010). Pestiviruses: How to outmaneuver your hosts. Vet. Microbiol..

[90] Tautz N., Tews B.A., Meyers G. (2015). The molecular biology of pestiviruses. Adv. Virus Res..

[91] Wang F.I., Deng M.C., Huang Y.L., Chang C.Y. (2015). Structures and functions of *Pestivirus* glycoproteins: Not simply surface matters. Viruses.

[92] Gottipati K., Acholi S., Ruggli N., Choi K.H. (2014). Autocatalytic activity and substrate specificity of the *Pestivirus* N-terminal protease N^pro^. Virology.

[93] Kappes M.A., Faaberg K.S. (2015). PRRSV structure, replication and recombination: Origin of phenotype and genotype diversity. Virology.

[94] Yun S.I., Lee Y.M. (2013). Overview: Replication of porcine reproductive and respiratory syndrome virus. J. Microbiol..

[95] Fang Y., Snijder E.J. (2010). The PRRSV replicase: Exploring the multifunctionality of an intriguing set of nonstructural proteins. Virus Res..

[96] Li Y., Zhou L., Zhang J., Ge X., Zhou R., Zheng H., Geng G., Guo X., Yang H. (2014). Nsp9 and Nsp10 contribute to the fatal virulence of highly pathogenic porcine reproductive and respiratory syndrome virus emerging in China. PLoS Pathog..

[97] Xie J., Zhou H., Cui J., Chen Y., Zhang M., Deng S., Zhou P., Su S., Zhang G. (2014). Inhibition of porcine reproductive and respiratory syndrome virus by specific siRNA targeting Nsp9 gene. Infect. Genet. Evol..

[98] Harfoot R., Webby R.J. (2017). H5 influenza, a global update. J. Microbiol..

[99] Bui C., Bethmont A., Chughtai A.A., Gardner L., Sarkar S., Hassan S., Seale H., MacIntyre C.R. (2016). A systematic review of the comparative epidemiology of avian and human influenza A H5N1 and H7N9—Lessons and unanswered questions. Transbound Emerg. Dis..

[100] Wang L., Wu A., Wang Y.E., Quanquin N., Li C., Wang J., Chen H.W., Liu S., Liu P., Zhang H. (2015). Functional genomics reveals linkers critical for influenza polymerase. J. Virol..

[101] Weigel T., Solomaier T., Wehmeyer S., Peuker A., Wolff M.W., Reichl U. (2016). A membrane-based purification process for cell culture-derived influenza A virus. J. Biotechnol..

[102] Zhong G., Le M.Q., Lopes T.J.S., Halfmann P., Hatta M., Fan S., Neumann G., Kawaoka Y. (2017). Mutations in the PA protein of avian H5N1 influenza viruses affect polymerase activity and mouse virulence. J. Virol..

[103] Fuller-Pace F.V. (2013). The DEAD-box proteins DDX5 (p68) and DDX17 (p72): Multi-tasking transcriptional regulators. Biochim. Biophys. Acta.

[104] Prockop S.E., Vatsayan A. (2017). Epstein-Barr virus lymphoproliferative disease after solid organ transplantation. Cytotherapy.

[105] Hau P.M., Tsao S.W. (2017). Epstein-Barr virus hijacks DNA damage response transducers to orchestrate its life cycle. Viruses.

[106] Kempkes B., Ling P.D. (2015). EBNA2 and its coactivator EBNA-LP. Curr. Top. Microbiol. Immunol..

[107] Sylvestersen K.B., Horn H., Jungmichel S., Jensen L.J., Nielsen M.L. (2014). Proteomic analysis of arginine methylation sites in human cells reveals dynamic regulation during transcriptional arrest. Mol. Cell Proteom..

[108] Kerr P.J., Liu J., Cattadori I., Ghedin E., Read A.F., Holmes E.C. (2015). Myxoma virus and the Leporipoxviruses: An evolutionary paradigm. Viruses.

[109] Chan W.M., Rahman M.M., McFadden G. (2013). Oncolytic myxoma virus: The path to clinic. Vaccine.

[110] Guerra S., Nájera J.L., González J.M., López-Fernández L.A., Climent N., Gatell J.M., Gallart T., Esteban M. (2007). Distinct gene expression profiling after infection of immature human monocyte-derived dendritic cells by the attenuated poxvirus vectors MVA and NYVAC. J. Virol..

[111] Moy R.H., Cole B.S., Yasunaga A., Gold B., Shankarling G., Varble A., Molleston J.M., ten Oever B.R., Lynch K.W., Cherry S. (2014). Stem-loop recognition by DDX17 facilitates miRNA processing and antiviral defense. Cell.

[112] Levrero M., Testoni B., Zoulim F. (2016). HBV cure: Why, how, when?. Curr. Opin. Virol..

[113] Iida-Ueno A., Enomoto M., Tamori A., Kawada N. (2017). Hepatitis B virus infection and alcohol consumption. World J. Gastroenterol..

[114] Seeger C., Mason W.S. (2015). Molecular biology of hepatitis B virus infection. Virology.

[115] Mani S.K.K., Andrisani O. (2018). Hepatitis B virus-associated hepatocellular carcinoma and hepatic cancer stem cells. Genes.

[116] Zhang Z., Kim T., Bao M., Facchinetti V., Jung S.Y., Ghaffari A.A., Qin J., Cheng G., Liu Y.J. (2011). DDX1, DDX21, and DHX36 helicases form a complex with the adaptor molecule TRIF to sense dsRNA in dendritic cells. Immunity.

[117] Kim T., Pazhoor S., Bao M., Zhang Z., Hanabuchi S., Facchinetti V., Bover L., Plumas J., Chaperot L., Qin J. (2010). Aspartate-glutamate-alanine-histidine box motif (DEAH)/RNA helicase A helicases sense microbial DNA in human plasmacytoid dendritic cells. Proc. Natl. Acad. Sci. USA.

[118] Mitoma H., Hanabuchi S., Kim T., Bao M., Zhang Z., Sugimoto N., Liu Y.J. (2013). The DHX33 RNA helicase senses cytosolic RNA and activates the NLRP3 inflammasome. Immunity.

[119] Zhang Z., Yuan B., Bao M., Lu N., Kim T., Liu Y.J. (2011). The helicase DDX41 senses intracellular DNA mediated by the adaptor STING in dendritic cells. Nat. Immunol..

[120] Miyashita M., Oshiumi H., Matsumoto M., Seya T. (2011). DDX60, a DEXD/H box helicase, is a novel antiviral factor promoting RIG-I-like receptor-mediated signaling. Mol. Cell Biol..

[121] Nikonov A., Mölder T., Sikut R., Kiiver K., Männik A., Toots U., Lulla A., Lulla V., Utt A., Merits A. (2013). RIG-I and MDA-5 detection of viral RNA-dependent RNA polymerase activity restricts positive-strand RNA virus replication. PLoS Pathog..

[122] Ranji A., Boris-Lawrie K. (2010). RNA helicases: Emerging roles in viral replication and the host innate response. RNA Biol..

